# Sleep rescues age-associated loss of glial engulfment

**DOI:** 10.1371/journal.pgen.1011999

**Published:** 2026-01-13

**Authors:** Jiwei Zhang, Elizabeth B. Brown, Evan Lloyd, Eshani Yeragi, Isabella Farhy-Tselnicker, Alex C. Keene

**Affiliations:** 1 Department of Biology, Texas A&M University, College Station, Texas, United States of America; 2 Department of Biological Science, Florida State University, Tallahassee, Florida, United States of America; Universidad de Valparaiso, CHILE

## Abstract

Neuronal injury due to trauma or neurodegeneration is a common feature of aging. The clearance of damaged neurons by glia is thought to be critical for maintenance of proper brain function. Sleep loss has been shown to inhibit the motility and function of glia that clear damaged axons while enhancement of sleep promotes clearance of damaged axons. Despite the potential role of glia in maintenance of brain function and protection against neurodegenerative disease, surprisingly little is known about how sleep loss impacts glial function in aged animals. Axotomy of the *Drosophila* antennae triggers Wallerian degeneration, where specialized olfactory ensheathing glia engulf damaged neurites. This glial response provides a robust model system to investigate the molecular basis for glial engulfment and neuron-glia communication. Glial engulfment is impaired in aged and sleep-deprived animals, raising the possibility that age-related sleep loss underlies deficits in glial function. To define the relationship between sleep- and age-dependent reductions in glial function, we used two complementary approaches to enhance sleep in aged animals and examined the effects on glial clearance of damaged axons. Both pharmacological and genetic induction of sleep restores clearance of damaged neurons in aged flies. Further analysis revealed that sleep restored post-injury induction of the phagocytic protein Draper to aged flies, fortifying the notion that loss of sleep contributes to reduced glial-mediated debris clearance in aged animals. To identify age-related changes in the transcriptional response to neuronal injury, we used single-nucleus RNA-seq (snRNA-seq) of the central brains from axotomized young and old flies. We identified broad transcriptional changes within the ensheathing glia of young flies, and the loss of transcriptional induction of autophagy-associated genes. We also identify age-dependent loss of transcriptional induction of 18 transcripts encoding for small and large ribosomal protein subunits following injury in old flies, suggesting dysregulation of ribosomal biogenesis contributes to loss of glial function. Together, these findings provide further support for a functional link between sleep loss, aging and Wallerian degeneration.

## Author summary

Aging is commonly associated with sleep disturbances and a decline in cognitive function, including impaired clearance of damaged neurites. Here, we use the fruit fly, *Drosophila melanogaster*, to investigate the relationship between age-related sleep loss and loss of glial capacity to remove damaged neurites. By employing both pharmacological and genetic methods to induce sleep in aged flies, we demonstrated that sleep restores glial function and enhances critical engulfment proteins in aged animals. Utilizing single-cell RNA sequencing, we identified key molecular pathways associated with age-related loss of glial function. This research paves the way for further investigations into how enhancing sleep could improve glial function and potentially mitigate age-related cognitive decline.

## Introduction

Sleep is a universal behavior that is critical for diverse aspects of brain function [1]. Chronic sleep disturbances are associated with numerous health consequences including neurodegenerative disease and cognitive decline [2,3]. Neurite damage due to apoptosis, trauma, or genetic factors is a common feature of aging, and the clearance of damaged neurons is thought to be critical for maintenance of proper brain function [4,5]. In the central and peripheral nervous systems, damaged neurites are cleared by Wallerian degeneration, a process where microglia or macrophages and Schwan cells are activated and engulf damaged neurites [6]. Despite the critical role of neurite clearance in maintenance of brain function and protection against neurodegenerative disease, surprisingly little is known about how life-history traits and environment modulate neurite clearance [7].

In flies and mammals, axotomy triggers Wallerian degeneration, where specialized glia engulf damaged neurites [8]. The genetic accessibility of the *Drosophila* olfactory system, combined with powerful genetic tools that allow for cell-type specific manipulation of gene expression, have made *Drosophila* a leading model to study injury-induced glial engulfment [9]. In *Drosophila,* olfactory ensheathing glia surrounds the antennal lobe then engulf the damaged olfactory neurons. Genetic screens in this system have identified numerous novel genetic factors and intercellular signaling pathways required for glial engulfment including *Stat92E, Draper, and Insulin Receptor* [10–14]. Further, numerous factors have been identified that function within neurons to signal neural injury including loss of the NAD+ synthase *Nmat1* and *Sarm* [9,12,15]. Modeling of Wallerian degeneration in other systems including neurons in the *Drosophila* wing, larval peripheral neurons, and mouse peripheral nerves confirm the mechanisms regulating Wallerian degeneration in the olfactory system are conserved in other *Drosophila* and mammalian neurons [16–18].

Glia are critical modulators of sleep duration, sleep-mediated neuronal homeostasis, and clearance of toxic substances during sleep [16–20]. Multiple mechanisms link glia to neurodegenerative disease including a role for clearance of Amyloid β (Aβ) by the glymphatic system and pruning of synapses by microglia in Alzheimer’s disease [21–23]. Recent findings suggest microglia function is inhibited by sleep deprivation in mouse models, raising the possibility that sleep directly impacts neuronal pruning and clearance within the brain [24]. Despite these bidirectional interactions between glia and sleep, surprisingly little is known about the mechanisms through which sleep deprivation impacts glial function, and how this contributes to neurodegenerative disease.

Growing evidence suggests sleep loss contributes to many of the functional deficits associated with aging [25,26]. Sleep loss shortens lifespan and accelerates many factors associated with aging, while quality and duration are reduced in old individuals [26,27]. In *Drosophila* aging, high calorie diets that accelerate aging, as well as acute sleep loss, all inhibit *draper* expression, resulting in reduced glial plasticity and clearance of injured neurites [19,28–30]. While the effects of age and sleep-related factors have largely been studied independently, both result in disrupted sleep, raising the possibility that reduced sleep quality contributes to age-related decline in glial function [31–33]. Examining the role of sleep on aging-associated loss of glial engulfment has potential to guide experimental approaches and therapeutic treatments for age-related decline in brain function.

Here, we examine the relationship between sleep and age-dependent changes on glial elimination of damaged neurites. Both pharmacological and genetic induction of sleep restore glial plasticity and Draper induction in aged flies. Further, the sleep-inducing effects of neural injury are absent in aged flies, revealing bidirectional interactions between sleep and aging. Together, these findings suggest sleep loss plays a critical role in age-dependent loss of glial cell function.

## Results

**Fig 1:**
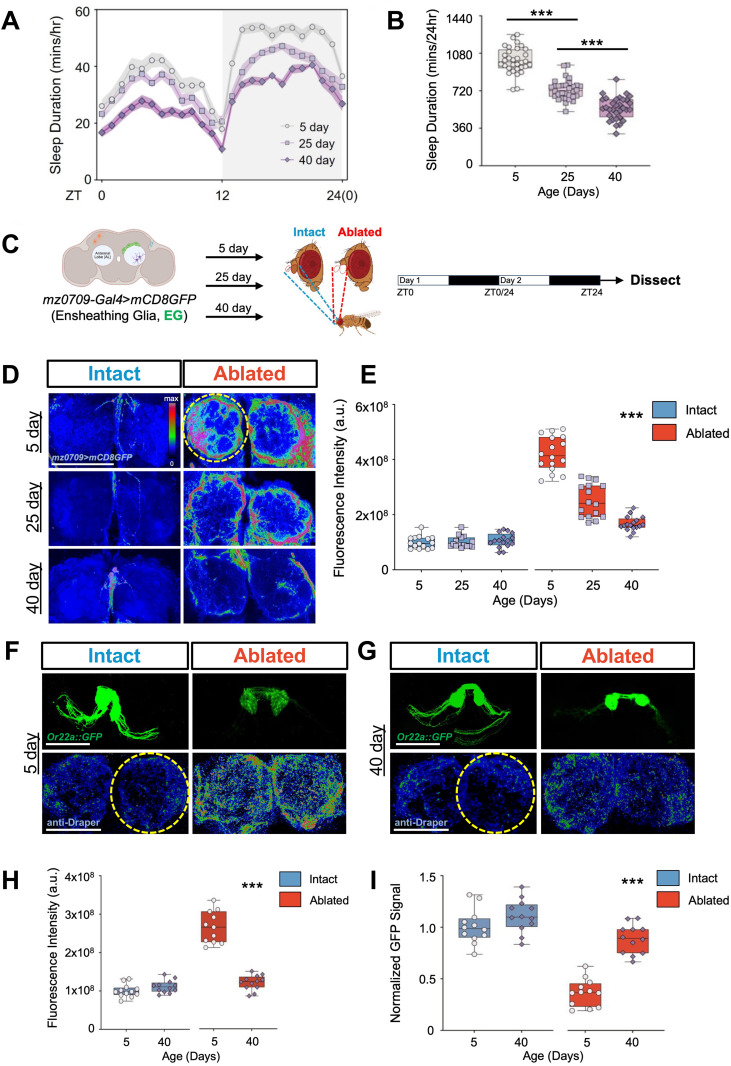
Glial membrane infiltration after neuronal injury is diminished in aged flies. (**A,B**) Sleep profiles and sleep duration of flies at 5 days (white, cycle), 25 days (mauve, square), and 40 days of age (purple, diamond). (**C**) Schematic of experimental design for the response of ensheathing glia (EG) to neuronal injury in young (5-day-old) and aged (25-day-old or 40-day-old) flies. (**D**) Extension of ensheathing glial processes following antennal ablation in the antennal lobes of *mz0709-Gal4* > *mCD8GFP* flies at 5, 25 and 40 days of age. The yellow circle indicates the region of the ensheathing glial membrane surrounding the antennal lobe that was quantified. (**E**) Quantification of glial membrane infiltration reveals a significant increase in ablated flies (red box) compared to unablated controls (blue box, two-way ANOVA, F_(2,84)_=77.69, P < 0.001) and a significant age-dependent decline in fluorescence intensity was observed at 25 days (P < 0.001) and 40 days of age (P < 0.001) compared to 5-day-old axotomized flies. (**F-G**) GFP signal labeled by *Or22a::GFP* and immunostaining for Draper in the antennal lobes of intact and ablated flies at 5- and 40-days of age. Hashed yellow circle represents the area of Draper quantification surrounding the antennal lobe and all fluorescence intensity were normalized to the 5-day intact controls. (**H**) Draper expression was significantly elevated in ablated 5-day-old flies compared to all other groups (P < 0.001, two-way ANOVA, F_(2, 21)_=94.79). (**I**) There was a significant reduction in GFP intensity in ablated 5-day old flies compared to all other groups (P < 0.001, two-way ANOVA, F_(1,44)_=21.06). Tukey’s multiple comparison tests: *P < 0.05; **P < 0.01; ***P < 0.001. Error bars indicate ± SEM. Scale bar denotes 50 μm. Fig 1C created in BioRender. Keene, A. (2026) https://BioRender.com/38r1thd.

Sleep duration and quality are reduced in aged flies [27,33–36]. To identify the specific timepoints when sleep duration and quality are reduced and to replicate previous findings that link aging to sleep loss, we compared sleep in flies aged 5, 25 and 40 days. There was a significant reduction in sleep in flies aged 25 days, and a greater loss at 40 days (Fig 1A and 1B). These changes in sleep duration are associated with reduced bout length and increased bout number, indicating reduced sleep quality (S1A-S1F Fig). Therefore, these timepoints were used to investigate the effects of age-related sleep loss on Wallerian degeneration.

Previous findings suggest that glial plasticity and Draper induction are impaired in aged flies [29]. To further examine the effects of aging on glial function we quantified Draper induction, neurite clearance and glial membrane infiltration in young and old flies. We ablated the antennae of flies aged 5, 25, and 40 days, expressing *mCD8:GFP* under control of the pan-glial driver *Repo* (S1G Fig) [9]. Glial membranes labeled with GFP robustly extend into the antennal lobes 24 hours following antennal ablation in young 5-day-old flies (S1H Fig). In contrast, glial membrane infiltration of the antennal lobes is diminished in flies injured at 25 days old, with an additional reduction observed in 40-day-old injured flies (S1I Fig). To confirm that the phenotypes observed were due to the recruitment of olfactory ensheathing glia, we selectively labeled these glia with *mz0709-Gal4* and quantified antennal lobe infiltration following antennal ablation (Fig 1C) [37]. Similar to results obtained with *Repo-Gal4*, the extension of glial processes into the antennal lobe following injury was reduced at 25 days and further diminished in ablated 40-day-old flies, while there were no age-dependent differences were noted in intact flies (Fig 1D and 1E). These findings confirm that the extension of ensheathing glial processes into the antennal lobe is impaired in aged flies.

Neuronal injury results in upregulation of the phagocytic protein Draper within olfactory ensheathing glia, leading to clearance of the damaged neurites [9]. To confirm that age-related deficits in glial plasticity are associated with the loss of Draper and reduced clearance of damaged neurites, we genetically labeled the olfactory receptor neurons (ORNs) using the *Or22a::GFP* transgene, which selectively labels ORNs from the antennae while excluding those from the maxillary palp [38]. The antennae of 5- or 40-day-old flies were ablated and the brains were subsequently immunostained for Draper 24 hours following injury. There were no differences in Draper levels in 5- and 40-day-old intact flies (Fig 1F-1G). Antennal ablation robustly induced Draper within the antennal lobes of 5-day-old flies, but not 40-day-old flies, confirming Draper induction is reduced in aged flies (Fig 1F-1H). Conversely, in 5-day-old flies, there was a significant reduction in *Or22a::GFP* signal, indicating robust glial elimination of damaged neurites (Fig 1F). *Or22a::GFP* signal was significantly higher in ablated 40-day-old flies compared to that in 5-day-old flies, revealing reduced clearance in aged flies (Fig 1F-1G and 1I). Together, these findings confirm that glial recruitment, Draper induction, and neurite engulfment in response to injury are reduced in 40-day-old flies.

**Fig 2:**
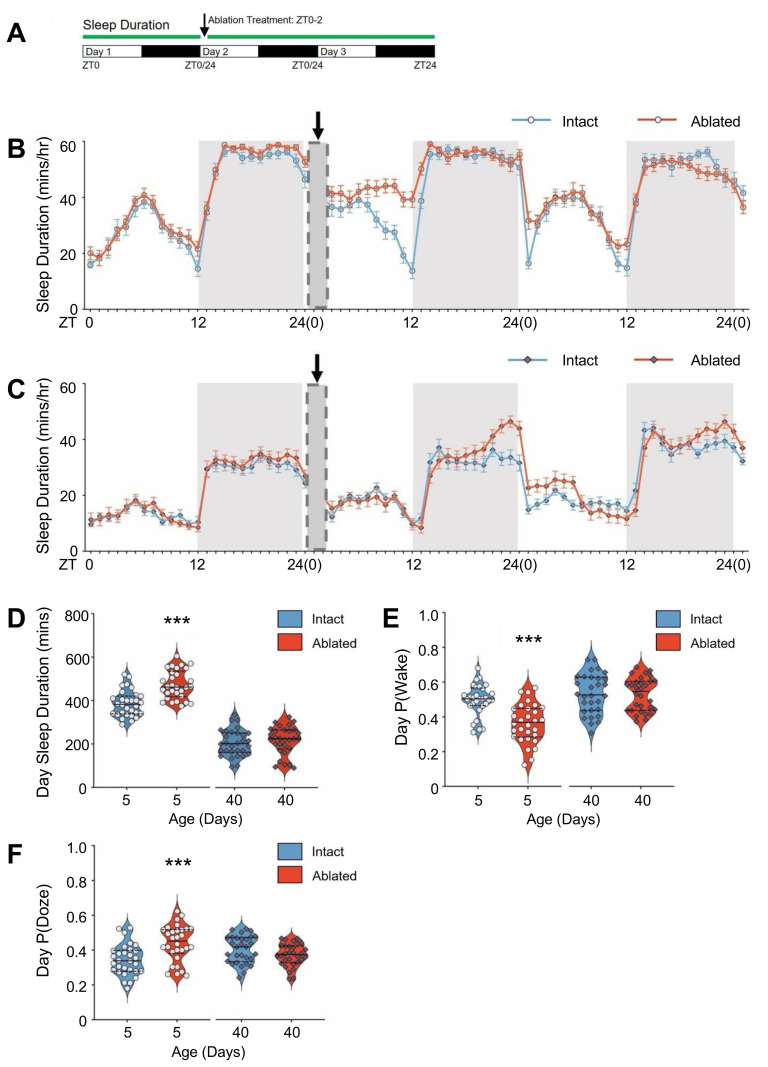
The sleep response induced by antennal ablation is disrupted in aged flies. (**A**) Diagram of experimental design for sleep response to neuronal injury in young (5-day-old) and aged (40-day-old) flies. (**B,C**) Sleep profiles prior to and following axonal injury in control unablated and ablated flies at 5 days (**B**) and 40 days (**C**) of age. Arrow denotes the time of antennal ablation, with the dark grey box (ZT0-2) representing a subsequent one-hour recovery period. White and gray shadows denote daytime and nighttime. (**D**) Daytime sleep duration was significantly increased in ablated flies following injury at 5 days of age but not at 40 days (P = 0.0002 and P = 0.4956, respectively). (**E**) Daytime wake propensity, P(Wake), was significantly reduced in 5-day-old flies but not in 40-day-old flies (P < 0.001 and P = 0.8644, respectively). (**F**) Daytime sleep propensity, (P)Doze, was significantly increased in 5-day-old flies following axotomy but not in 40-day-old flies (P = 0.0003 and P = 0.0843, respectively). *P < 0.05; **P < 0.01; ***P < 0.001. Error bars indicate ± SEM.

Injury and immune activation are associated with increased sleep in *Drosophila* [18,33]. In flies, sleep is increased immediately following antennal ablation, providing a system to examine the effects of aging on neural injury response [18]. To examine the effects of aging on sleep following injury, we quantified sleep immediately following injury in 5- and 40-day-old flies (Fig 2A). In agreement with previous findings, 5-day-old antennal ablated flies slept significantly more than intact controls (Fig 2B) [18]. Conversely, there was no increase in daytime sleep following ablation in 40-day-old ablated flies (Figs 2C, 2D, and S2A). In 40-day-old flies there was an increase in nighttime sleep 12 hours from injury, suggesting a reduced and delayed response (S2A Fig). At day two following ablation sleep did not differ from controls for both young and old flies, revealing the immediate effects of injury do not result in long-lasting changes (Fig 2B and 2C). The increased sleep following injury in 5-day-old flies is due to longer bout lengths, consistent with greater sleep consolidation (Figs 2D, and S2D-S2K).

We applied a Markov model that determines propensity to remain asleep, which serves as an indicator of sleep depth [37]. Following injury, sleep propensity, P(Doze), was elevated and wake propensity, P(Wake), was reduced in 5-day-old flies, but not in 40-day-old flies, supporting the notion that sleep drive is diminished in aged flies (Figs 2E, 2F, and S2B-S2C). Furthermore, ablation induced an increase in average bout length during the day but not at night in 5-day-old flies, whereas no such effect was observed in 40-day-old flies (S2F-S2G and S2J-S2K Fig). Therefore, aging disrupts sleep-induction following injury. These findings support the idea that both reduced levels of basal sleep, and impaired sleep induction following injury contribute to age-related loss of glial activation and Wallerian degeneration.

Draper is required for the clearance of damaged neurites, but its role in sleep regulation has not been investigated. To determine whether Draper also regulates sleep, we selectively knocked down draper in all glia using Repo-GAL4, or specifically in ensheathing glia using mz0709-GAL4 **o**r R56C01-GAL4. In all three cases, knockdown flies slept less than those carrying either GAL4 or draper-RNAi alone (S3A Fig). To test whether Draper is required for post-injury sleep, we injured mz0709-GAL4 > draper-RNAi flies and measured sleep immediately following antennal ablation. Knockdown of draper in ensheathing glia abolished post-injury sleep, and injured flies were indistinguishable from intact draper knockdown controls (S3B and S3C Fig). We next asked whether Draper is required for injury-induced changes in waking activity. In mz0709-GAL4 > draper-RNAi flies, there was no difference in waking activity between intact and ablated animals (S3D Fig). Together, these findings demonstrate that Draper functions in ensheathing glia to promote basal sleep and is essential for the post-injury sleep response.

**Fig 3:**
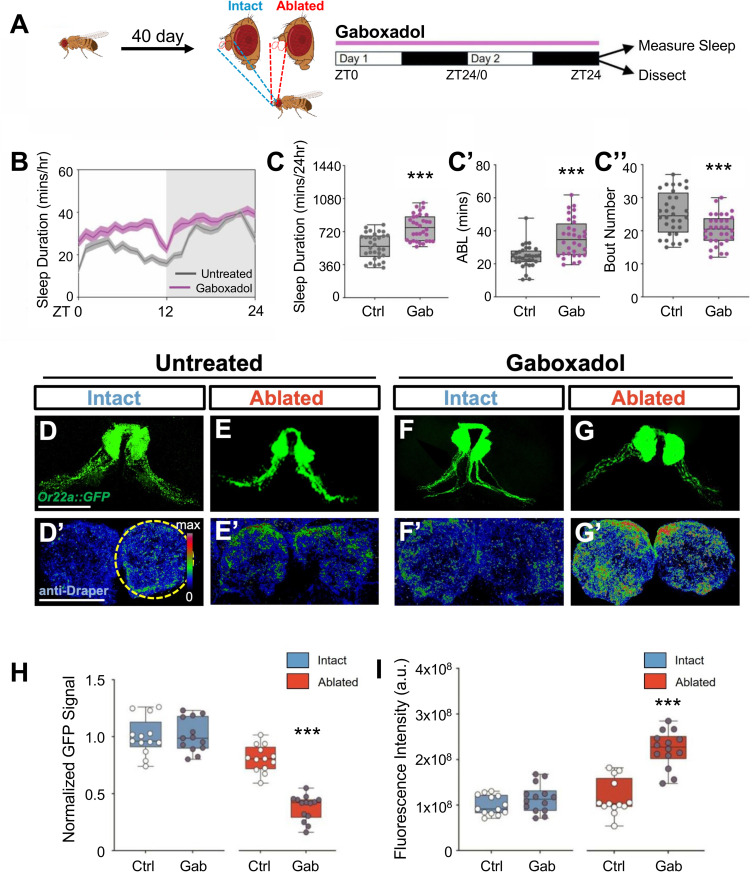
Gaboxadol-induced sleep mediates the restoration of glial process extension in aged flies. (**A**) Diagram of gaboxadol treatment and subsequent behavior monitoring and sample preparation. (**B**) Sleep profile of 40-day untreated and gaboxadol treated flies. (**C**) Total sleep and average bout length (ABL, **C’**) was significantly increased by gaboxadol treatment in 40-day-old flies (P < 0.0001 and P < 0.0001, respectively), while bout number (**C”**) significantly decreased (P < 0.001). (**D-G**) GFP signal labeled by *Or22a::GFP* and (**D’-G’**) immunostaining for Draper in the antennal lobes of gaboxadol treated and untreated intact or axotomized 40-day-old flies. Scale bar denotes 50 μm. (**H**) GFP quantification revealed no difference between gaboxadol treated (dark dots) and untreated control (white dots) intact flies (P = 0.8833). There was a significant reduction in GFP intensity in ablated gaboxadol treated flies compared to all other groups (P < 0.001). (**I**) Draper levels were significantly elevated in ablated 40-day-old gaboxadol treated flies compared to all other groups (P < 0.001). ***P < 0.001 by one-way ANOVA followed by Turkey’s post-hoc tests. Error bars indicate ± SEM. Fig 3A created in BioRender. Keene, A. (2025) https://BioRender.com/ejp67b0.

The engulfment phenotypes observed in aged flies phenocopy those previously reported in response to acute sleep deprivation, raising the possibility that sleep deficits contribute to the age-related decline in glial clearance of damaged neurites [19]. To examine whether age-associated sleep loss contributes to loss of ensheathing glial response to injury we sought to increase sleep and measure the effect on clearance of damaged neurons. We pharmacologically enhanced sleep in young and old flies following antennal ablation and measured the effects on glial plasticity and axon engulfment [35,38]. Five- or 40-day old flies were fed the sleep-inducing GABA agonist gaboxadol for 48 hours following injury, after which Wallerian degeneration was quantified (Figs 3A and S4A) [38]. Gaboxadol treatment significantly increased sleep in both young and old flies by increasing the average bout length (Figs 3B, 3C, S4B and S4C). Signal from *Or22a::GFP*-labeled neurites was significantly reduced in ablated 5- and 40-day-old flies treated with Gaboxadol compared to solvent treated controls revealing that pharmacological induction of sleep restores glial elimination to old flies (Figs 3D, 3E, S4D and S4E). In both 5- and 40-day-old flies there was no effect of gaboxadol treatment on unablated controls, confirming that gaboxadol does not promote axonal engulfment in uninjured controls (Figs 3F and S4F). Similarly, Draper levels were elevated in both 5 and 40-day-old ablated flies treated with gaboxadol compared to age-matched untreated controls, while there was no effect of gaboxadol on Draper levels in flies with intact antennae (Figs 3F’, 3G’, 3I, S4F’, S4G’, and S4I). Therefore, feeding flies gaboxadol, which reportedly increases sleep, restores age-related deficits in neurite clearance and glial activation.

**Fig 4:**
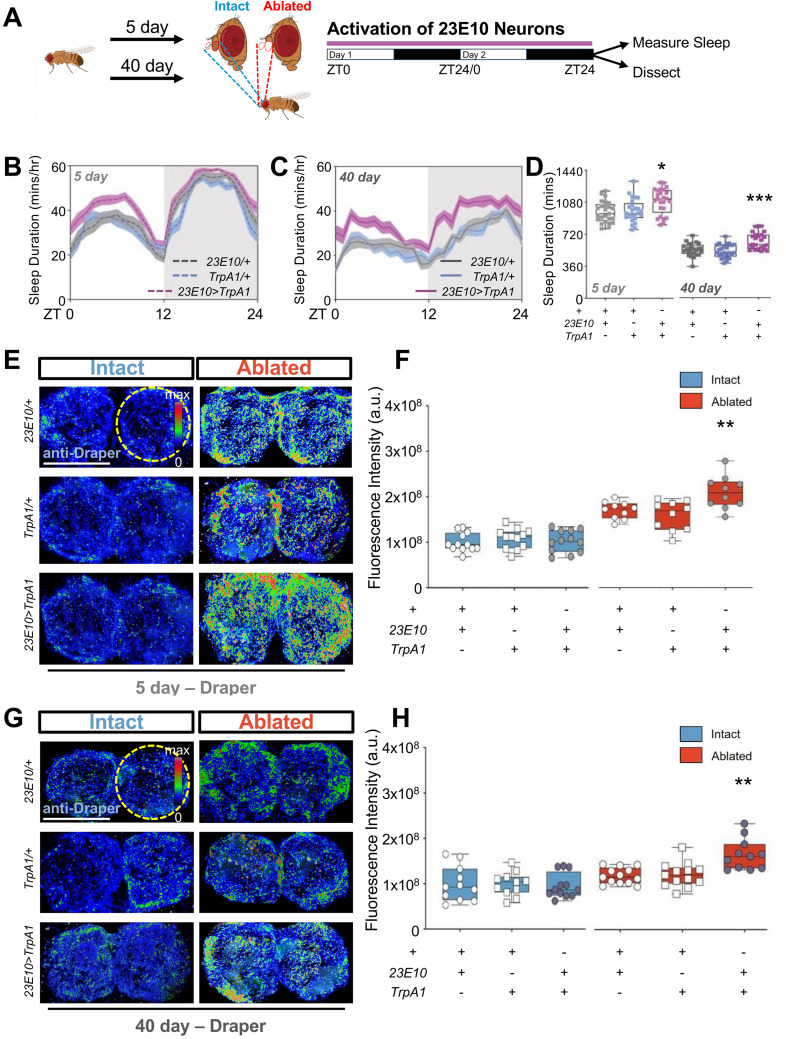
Genetically induced sleep drives sleep-dependent glial remodeling and Draper recruitment in aged flies post neuronal injury. (**A**) Diagram of sleep induction by thermogenetic activation of 23E10 neurons and subsequent sleep measurement and quantification of glial clearance. (**B-D**) Elevated temperature significantly induced sleep in 5-day-old (**B**) and 40-day-old (**C**) *23E10-Gal4* > *TrpA1* flies compared to *TrpA1*/*+* or *23E10-Gal4*/ *+* controls. (**D**) Total sleep was significantly increased after two-day thermo-activation of 23E10 neurons in both 5-day-old (P = 0.0022) and 40-day-old (P = 0.0001). (**E**) Immunostaining for Draper levels in the antennal lobes in control (*23E10-Gal4*/ + ,*TrpA1*/+) and experimental (*23E10-Gal4* > *TrpA1*) 5-day-old flies. (**F**) There were no differences in Draper levels in intact flies (P = 0.8455), while Draper levels were significantly elevated in *23E10-Gal4* > *TrpA1* ablated flies, compared to ablated flies without sleep induction (P = 0.0072). (**G**) Immunostaining for Draper levels in the antennal lobes in control (*23E10-Gal4*/ + ,*TrpA1*/+) and experimental (*23E10-Gal4* > *TrpA1*) 40-day-old flies. (**H**) There were no differences in Draper levels in intact flies (P = 0.7409), while Draper levels were significantly elevated in *23E10-Gal4* > *TrpA1* ablated flies, compared to ablated flies without sleep induction (P = 0.0003). Scale bar denotes 50 μm. *P < 0.05, **P < 0.01, ***P < 0.001. Error bars indicate ± SEM. Fig 4A created in BioRender. Keene, A. (2025) https://BioRender.com/ejp67b0.

It is possible that the effects of gaboxadol on glial activation and neurite engulfment are due to activation of GABA signaling rather than induction of sleep per se. To differentiate between these possibilities, we genetically activated R23E10 neurons that label sleep promoting neurons in the brain and/or the ventral nerve cord [39–41] and measured the effect on clearance of damaged neurites. Briefly, we ablated the antennae of flies expressing the thermosensitive cation channel *TrpA1* in R23E10 neurons (*R23E10-Gal4* > *TrpA1*), and increased the temperature from 22°C to 29°C for 48 hours following injury. Following sleep induction, brains were immunostained for Draper levels (Fig 4A). Consistent with previous findings in young flies, thermogenetic activation of 23E10 neurons increased sleep in 5- and 40-day-old flies (Figs 4B–4D and S5A-S5E) [39]. Induction of sleep enhanced Draper levels in 5-day-old flies compared to genetic controls (Fig 4E and 4F). There was no effect of sleep induction on Draper levels in intact 5-day-old flies, suggesting the effects of sleep on Draper is specific to post-injury response (Fig 4F). Similarly, in 40-day-old flies, genetic induction of sleep increased Draper levels in ablated flies but not in unablated controls (Fig 4G and 4H). Together, these findings confirm that genetically inducing sleep is sufficient to restore post-injury Draper levels in aged flies.

**Fig 5:**
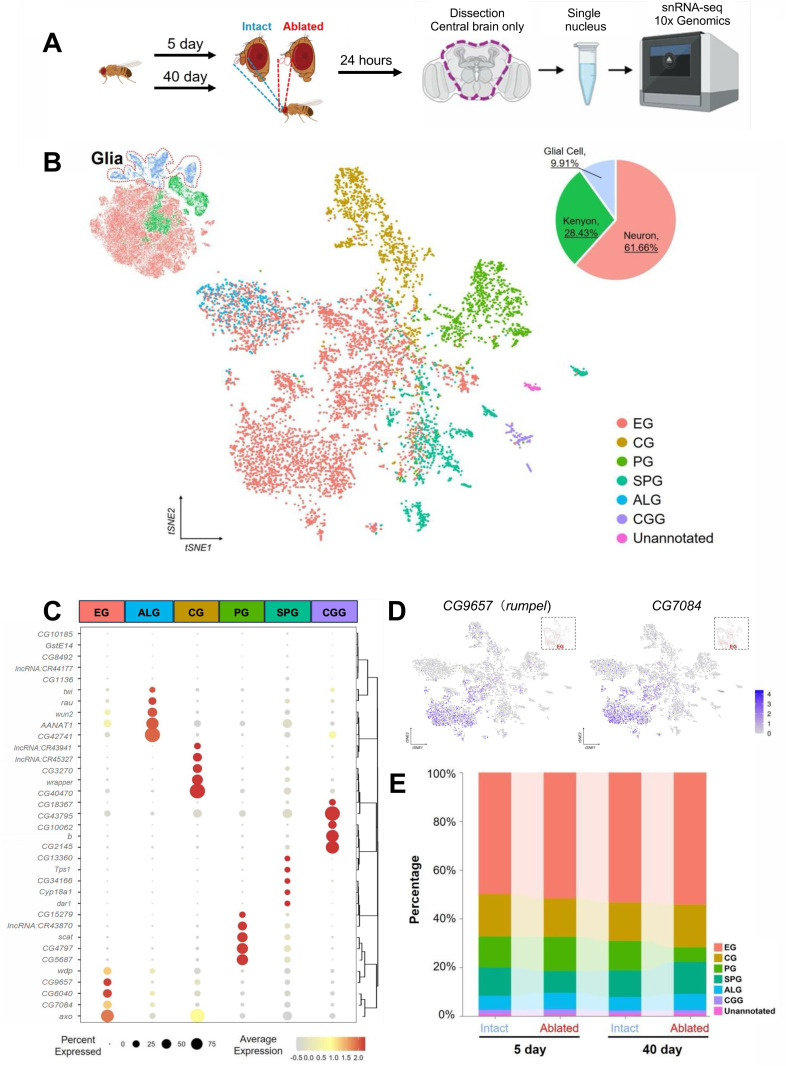
snRNA-seq of central brains in young and aged intact or ablated flies. (**A**) Schematic of tissue collection and snRNA-seq workflow. Axotomy of the third segment of antenna was performed for the adult flies at 5- or 40-days of age. After 24 hour of recovery following the axotomy, the central brains of intact or ablated flies were dissected and dissociated to collect single nuclei. (**B**) t-distributed Stochastic Neighbor Embedding (t-SNE) visualization of the 7 distinct subclusters of glial cells from the integrated nuclei of 5-day intact or ablated and 40-day intact or ablated pooled replicate samples. Each color corresponds to a unique cluster, and each dot represents a single nucleus. EG, ensheathing glia; CG, cortex glia; PG, perineurial glia; SPG, subperineurial glia; ALG, astrocyte-like glia; CGG, chiasm giant glia; Unannotated, unannotated glial types. (**C**) Dotplot of marker genes for each glial subtype that were used for annotation. (**D**) t-SNE visualization of selective EG marker genes, *rumpel* (left) and *dSLC22a3* (right), on glia cluster. (**E**) Percentage of glial subtypes among four samples. Fig 5A created in BioRender. Keene, A. (2025) https://BioRender.com/ejp67b0.

Numerous genetic pathways have been identified as contributing to glial activation and increased Draper levels following axotomy, yet much less is known about how aging impacts glial function [8]. To identify age-dependent changes in ensheathing glia we performed snRNA-seq on the central brains of both young and old flies under intact and axotomized conditions (Fig 5A). We dissected 120–140 central brains of intact or ablated flies at 5 and 40 days of age. This yielded at least 295k nuclei for each of the four experimental groups, resulting in 68,361 nuclei that met the criteria for inclusion in analysis (see Materials and Methods). There were no significant differences in nuclei number between any of the groups tested, suggesting consistency between sample collections. We were able to classify nuclei into diverse types of brain cell types based on known markers (Figs 5B–5C and S6A-S6D). Clustering analysis revealed six distinct populations and chiasm giant glia, and one unannotated group (Figs 5B-5D and S6E). The total number, and types of glia did not including cortex glia (CG), ensheathing glia (EG), astrocyte-like glia (ALG), perineural glia (PG), subperineural glia (SPG) and chiasm giant glia, and one unannotated group (Figs 5B–5D and S6E). The total number, and types of glia did not vary significantly across experimental conditions, supporting the notion that loss of neurite engulfment in aged flies is not due to an overall loss of ensheathing glia (Figs 5E and S6F).

**Fig 6:**
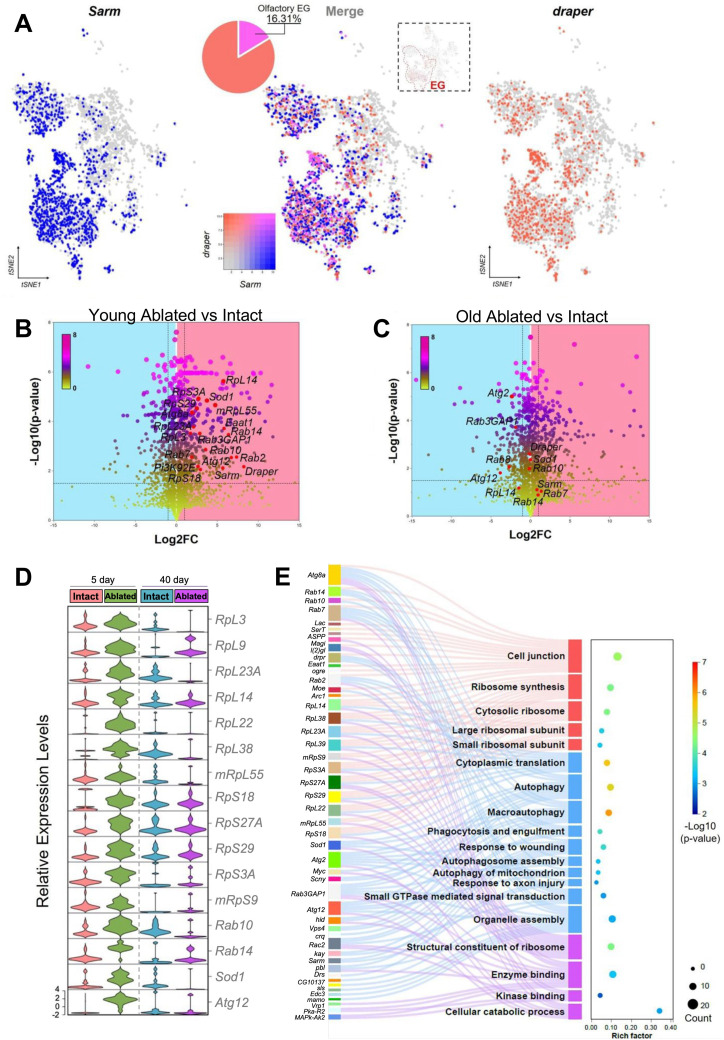
Differential gene expression analysis of olfactory ensheathing glia in young and aged intact or ablated flies. (**A**) t-SNE visualization of *Sarm* and *draper* positive olfactory ensheathing glial nuclei. Nuclei that express *Sarm* (*Sarm*^+^) are highlighted in blue, while nuclei that express *draper* (*draper*^+^) are highlighted in red. *Sarm* and *draper* co-expressed olfactory EG were merged in pink with a color box indicated the expression levels of *Sarm* or *draper* in ensheathing glial cell. (**B**) Volcano plot showing the differential expressed genes (DEG) in *Sarm*^*+*^ and *draper*^*+*^ olfactory ensheathing glial nuclei between 5-day ablated and intact controls. (**C**) Volcano plot showing the DEG in *Sarm*^*+*^ and *draper*^*+*^ olfactory ensheathing glial nuclei between 40-day ablated and intact groups. The dashed lines represent the thresholds for significance. Genes with significant differential expression were highlighted in dark colors, while non-significant genes are shown in lime green. Blue background was used for downregulated genes and pink background was used for highlighting upregulated (activated response to neural injury) genes. Candidate genes were highlighted in red dots. (**D**) Gene expression levels of candidate genes across each treatment. (**E**) Ribosomal protein subunit genes and autophagy or phagocytosis related pathways were significantly enriched in 5-day-old flies post neural injury.

To identify genes that are transcriptionally regulated in response to neural injury in young and old flies, we compared gene expression across experimental groups within the ensheathing glia cluster. We first sought to specifically identify the olfactory ensheathing glia based on the expression of *repo* and the Toll-1 receptor *Sarm*, and *draper*, all of which are highly expressed in olfactory ensheathing glia post neural injury [42–44]. We specifically analyzed the ensheathing glia group. Of these, 16.31% co-expressed *draper* and *Sarm*, and were therefore defined as olfactory ensheathing glia (Figs 6A and S7A). This subset was further examined for differential gene expression.

To determine the differences in gene expression between young and old individuals in response to neuronal injury, we compared the transcriptional changes in genes between the young and old groups of olfactory ensheathing glial nuclei, using Seurat’s FindMarkers function. In the comprehensive gene expression analysis, we observed distinct patterns of differential gene regulation between intact and ablated groups in young and old cohorts separately. In the young cohort, the ablated group showed a significant divergence, with 927 genes upregulated and 670 genes downregulated compared to the intact group (Figs 6B and S7B). This robust response suggests a pronounced compensatory mechanism for engulfment of damaged neurites or altered developmental trajectory in response to ablation. Conversely, in the aged cohort, the ablated flies displayed a distinct profile, with 877 genes significantly upregulated, of which 325 overlapped with those upregulated in the young group (Figs 6C and S7B). This overlap indicates a subset of genes that maintain their function throughout the lifespan in response to neuronal injury. Among the 602 uniquely upregulated genes in the young, ablated group, we identified key candidates, including autophagy-related genes *Atg12*, *Atg8a*, *Rab10*, *Rab14*, *Rab7*, and *Rab3GAP1*, along with several ribosomal subunit genes, as well as *draper* and *Sarm*. These genes may play pivotal roles in efficient neurite engulfment within 24 hours post-injury and warrant further investigation regarding their functional implications in development and aging (Fig 6B-6D).

Next, we performed pseudobulk differential expression analysis on olfactory EG cell cluster from the raw count data and subsequently used z-score clustering expression to identify different patterns of gene expression across all four experimental groups (S7C Fig). We identified 1,356 genes in cluster 2 that were selectively upregulated in young flies following ablation, but not in old flies. By conducting these two analyses and overlapping the 602 genes with the genes in cluster 2, we specifically focused our analysis on the genes that were upregulated in the young cohort but not in aged flies following neural injury (S7B and S7C Fig). The candidate genes mentioned above, including a set of ribosomal subunit genes and several autophagy-related genes involved in phagocytosis and engulfment pathways, all exhibit this expression pattern (Figs 6D, 6E and S7C). Strikingly, seven large ribosomal protein subunit (RpL) transcripts and five small ribosomal protein subunit (RpS) transcripts were significantly upregulated following ablation in young flies, but not in old ablated flies (Figs 6D and S7D). These genes present numerous potential candidate regulators of glial recruitment and the injury response, highlighting the role of increased ribosomal synthesis following injury (Figs 6E and S7D). Gene ontology analysis revealed that genes related to both ribosome function and autophagy are equally important for cellular processes following injury, specifically associated with Wallerian degeneration (Fig 6E). Together, these analyses identify numerous genes that are transcriptionally regulated by both neural injury and aging, providing candidate regulators for the age-dependent loss of Wallerian degeneration.

## Discussion

Here, we show a link between reduced sleep and age-dependent loss of Wallerian degeneration in *Drosophila*. Both aging and acute sleep loss are associated with reduced brain function including changes in structural plasticity, long-term potentiation, memory, and elevated oxidative stress levels [25,27,30,45]. Previous work in mice and flies has shown that both aging and sleep loss impair glial engulfment, but the relationship between sleep loss and aging has been unclear [29,19]. Here, we show that pharmacological and genetic induction of sleep restores induction of Draper and glia-mediated engulfment to aged flies.

Age-related loss of glial function is associated with many aspects of declining brain function. In flies and mammals, the clearance of debris and toxic substances from the brain is critical for healthy aging. In mammals, both the overactivation and reduced function of microglia is associated with neurodegeneration [24,46,47], while the glymphatic systems play a critical role in regulating the clearance of amyloid beta from cerebral spinal fluid [22]. In *Drosophila,* loss of the ensheathing glia is associated with lipid accumulation and reduced lifespan, while genetically increasing ensheathing glia number is protective in a model of Alzheimer’s disease [48]. Further, loss of glial function accelerates age-related changes in memory [49]. These findings suggest ensheathing glia are critical for healthy aging. Here, we find that age-related sleep loss contributes to reduced glial function. These findings raise the possibility that loss of glia function may contribute to the negative impacts of aging.

There are many functional parallels between *Drosophila* and mammalian glia and their roles in sleep regulation. Both astrocytes and microglia are essential for normal brain function in animals ranging from flies to mice. These effects appear to be highly conserved because astrocytic-like and microglia-like ensheathing glia also regulate sleep in *Drosophila* [16,50,51]. In some cases, glia appear to impact global brain function, such as regulation of synaptic strength and neural excitability, while in other cases glia appear to modulate levels of specific sleep transmitters or neural circuits [52–55]. It is possible that the effects of sleep and aging in modulating glia function extend beyond olfactory ensheathing glia, to other types of glia in the brain. Supporting this notion, our snRNA-seq data identified *Sarm*- and *draper*-positive glial cells in the CG, PG, SPG and ALG subtypes (S7E-S7H Fig). Future studies examining the effects of aging on astrocyte-like glia, cortex glia, and non-olfactory ensheathing glia is critical to understand whether aging and sleep loss impact all types of glia or are specific to the engulfing properties of olfactory ensheathing glia.

In these experiments, we used two complementary methods to increase sleep: activation of neurons labeled by the GAL4 driver 23E10 and administration of the GABA receptor agonist gaboxadol. While both approaches restored Draper induction following injury in aged flies, each approach may not fully recapitulate natural sleep. Specifically, 23E10 activation and gabaxadol differ dramatically in their effects on global neural activity and transcriptional regulation [35,56]. Moreover, although many studies have characterized activation of 23E10 neurons as sleep-promoting, others report no detectable sleep phenotype with this manipulation [57,40]. Numerous additional sleep-promoting neurons have been identified including RDL neurons, DPM neurons, and subsets of mushroom body neurons [58–60]. Furthermore, alternative experimental manipulation, including mechanical stimulation such as vibration to induce sleep offer further avenues to test the hypothesis that sleep restores age-dependent loss of glial function [61]. Incorporating these additional approaches will be critical for rigorously establishing whether sleep is sufficient to rescue age-related declines in glial responsiveness.

*Drosophila* increase sleep immediately following ablation injury to the antennae or wing suggesting this is a naturally occurring mechanism to promote recovery and glial-mediated engulfment [18]. The degree of post-injury sleep is thought to directly result from synaptic pruning, because expression of neuroprotective factors in the olfactory neurons blunt sleep following injury [18]. Here, we show that post-injury sleep is reduced in aged flies, consistent with the notion that pruning promotes sleep following injury. These findings raise the possibility that bi-directional interactions between neurite clearance and sleep regulation are both impacted by aging. The effects were greatest in the first 12 hours following injury. It is important to note that the analysis in this manuscript was limited to activity-based monitoring. Additional investigations, such as assessments of arousal threshold, whole-brain imaging, or whole-body metabolic rate, are critical to determine the specific type of sleep induced by injury [35,62,63]. Nevertheless, the findings raise the possibility that greater upregulation of neuroprotective factors contribute to the blunted sleep response. Because ORN cell bodies are removed in the process, we are unable to address this using our snRNA-seq protocol. However, future work specifically examining interactions between post-injury sleep induction and neuroprotective factors in aged flies may be informative.

Transcriptional regulation in glia is modulated by sleep, circadian timing, and age [64,65]. These molecular factors directly impact glial function including clearance of debris, regulation of neurotransmitter release, and secretion of sleep-regulating factors [51,66]. For example, insulin signaling promotes sleep and activates ensheathing glia phagocytotic mechanisms in *Drosophila*, while diet induced insulin resistance inhibits neurite clearance, revealing a critical role for insulin signaling in debris clearance [13,28]. To identify new factors that contribute to age related changes in sleep we performed snRNA-seq in the central brains of old and young flies with ablated antennae. We find numerous genes previously known to be under transcriptional regulation including *draper* and the autophagy regulators *Atg8a* and *Rab5* are not upregulated following axotomy in aged flies. Altered autophagy is linked to sleep, as autophagosome levels accumulate during periods of wakefulness. Sleep loss is associated with reduced proteostasis, ultimately leading to neurodegeneration [60,61].

Our findings indicate that aging inhibits induction of ribosomal subunit transcription in glia, which is consistent with studies ranging from *C. elegans* to humans that reveal an age-associated loss of ribosomal protein subunits [67,68]. Consequently, the loss of ribosomal biogenesis may inhibit the structural and functional plasticity required to engulf aging neurites. It is possible that the injury-induced ribosomal subunits are generally required for enhanced translation following injury, or that these subunits confer unique functions. In *Drosophila*, selective loss of individual ribosomal subunits is associated with defects in development and cellular function [69–71] Furthermore, the TOR pathway has been implicated more generally in age-associated loss of ribosomal biogenesis including reduced TOR signaling [72]. Therefore, it is possible that pharmacological enhancement of ribosomal biogenesis through these pathways has potential to ameliorate the cellular, and possibly functional, effects of age-related sleep loss [73].

Ensheathing glia are critical for maintaining brain health, and preventing their age-dependent loss extends lifespan in Alzheimer’s model *Drosophila* [48]. A central question is how the transcriptional changes identified in aged flies alter the physiology of ensheathing glia, and whether these changes in turn influence longevity. Multiple studies have applied Ca² ⁺ imaging to characterize physiological changes across diverse classes of glia [74]. In ensheathing glia specifically, Ca² ⁺ activity is highly dynamic and responsive to shifts in neurochemistry and behavior [75]. Given that optogenetic activation of ensheathing glia promotes sleep, age-related changes in brain-wide neurochemistry or transcriptional regulation within glia could have important consequences for both glial function and behavior [75]. Future studies examining the impact of ribosomal dysregulation on ensheathing glia physiology will be particularly valuable for pinpointing gene-specific contributions to the age-dependent decline in glial function.

Taken together, our findings demonstrate a role for sleep in the age-related loss of glial-mediated phagocytosis. Sleep is sufficient to restore glial membrane plasticity, facilitate the induction of Draper, and enhance neurite engulfment in aged flies. These findings provide a framework for examining the effects of sleep on glial-mediated phagocytosis and how sleep and aging impact glial function more broadly. Given that many aspects of glial function, including their roles in regulating brain health, are conserved across species from flies to mammals, this system has the potential to advance our understanding of changes in glial function throughout the lifespan.

## Materials and methods

### Fly stocks and maintenance

Flies were reared and maintained on a 12:12 light–dark cycle in humidified incubators at 25°C and 65% humidity (Percival Scientific, Perry, IA, USA). The following fly lines were obtained from the Bloomington *Drosophila* Stock Center: *UAS-mCD8GFP* (BDSC #32186; [76]), *Repo-Gal4* (BDSC #7415; [77]), *OR22a::GFP* (BDSC #52620; [78]), *23E10-Gal4* (BDSC #49032) and *UAS-TrpA1* (BDSC #26263; Paul Garrity). The background control line used in this study was *w*^*1118*^ (BDSC # 5905; [79]) and all experimental stocks were either generated in this background or outcrossed to *w*^*1118*^ for a minimum of six generations prior to testing. Unless indicated otherwise, mated females reared on standard *Drosophila* food media (Bloomington Formulation, Nutri-fly, #66–113, Genesee Scientific) at the 4–5 day of age were used for all experiments. For the aging experiments, flies were isolated one day post-eclosion and sorted by sex into vials containing approximately 30 flies each. They were then transferred to new vials three times per week until reaching the indicated age. Unless otherwise noted, all experiments were performed using mated female flies.

### Fly axotomy

Fly axotomoy was performed as previously described [19]. Flies of the indicated age and genotype were gently anesthetized on CO_2_ for less than one minute at ZT0–2. Next, using forceps, the third segments of both antennae were removed. Controls received the same treatment without ablation. Unless indicated otherwise, control and ablated flies were maintained on standard food for 24 hours prior to dissection.

### Drug treatment

Gaboxadol (4,5,6,7-retrahydroisoxazolo [5,4-*c*] pyridin-3-ol hydrochloride, THIP hydrochloride; Sigma Aldrich, St. Louis, MO, USA, #T101) was dissolved in dH_2_O at 1 mg/ml, then mixed to 0.01 mg/ml in cooled, standard fly food, as previously described [38,63]. Axotomized and intact control flies were placed on gaboxadol-laced food immediately following axotomy, where they remained for the duration of the experiment.

### Measurements of sleep behavior

Sleep behavior was measured using the *Drosophila* Activity Monitoring (DAM) system (Trikinetics, Waltham, MA), which detects activity by monitoring infrared beam crossings of individually housed flies. The number of beam-breaks is used to determine the amount of sleep, along with associated sleep metrics, by identifying 5-min bouts of quiescence using the *Drosophila* Sleep Counting Macro [80,81]. All experiments were performed in incubators at the same temperature and humidity mentioned above. For sleep experiment in aging flies, flies of the indicated age were briefly anesthetized with CO_2_ and loaded individually in DAMs tubes with standard fly food. Flies were acclimated for a minimum of 24 hours prior to the start of behavioral analysis. Measurements of sleep were then measured over a 24 hour period starting at ZT0. For sleep experiments in axotomized flies, sleep was measured for 24 hour as described in aging flies, however at ZT 0–2 the following day, individual flies were removed, axotomized, and then returned to their respective tube and monitor, in which sleep was recorded for the subsequent 48 hours post axotomy. Control flies received the same treatment without ablation. For sleep-induction experiments, sleep was measured in flies fed 0.01 mg/ml Gaboxadol. For *23E10-Gal4* activation experiments, sleep was measured at the permissive temperature of 29°C.

### Immunohistochemistry and imaging

Brains of the indicated age and treatment were dissected in cold phosphate buffer solution (PBS) and fixed in 4% paraformaldehyde in PBS containing 0.5% Triton-X100 [PBT], for 40 minutes at room temperature (RT). After three washes (15 min each) with PBT and rinsing overnight at 4°C, the brains were incubated with relevant primary antibody, anti-GFP (1:1500; #PA1-980A, Fisher Scientific) or anti-Draper 5D14 (1:500; #draper, AB_2618105, Developmental Studies Hybridoma Bank), for 48 hr at 4°C with rotation. After three 15-min washes in PBT at RT, the brains were incubated with secondary antibody (Alexa Fluor 555 donkey anti-mouse IgG, Invitrogen, Carlsbad, CA, USA, #A31570; Alexa Fluor 488 goat anti-rabbit IgG, Invitrogen, Carlsbad, CA, USA, #A11008) for 90 min at RT, and then washed three times (30 min each) in PBT, followed by a final overnight wash in PBT. Brains were mounted in Vectashield (H1000, Vector Labs, Burlingame, CA, USA). Fluorescence images were acquired on a Nikon AX R Laser-Scanning Confocal Microscope (Nikon Corporation, Tokyo, Japan). Brains were imaged using the 561 nm and 488 nm lasers sequentially, with imaging settings (e.g., laser power, gain, and zoom) kept constant throughout the entire experiment. Image stacks were acquired using the ND Sequence Acquisition function, imaging from the above to below the antennal lobes using a 2-μm step size.

### Quantification of fluorescence intensity

Quantification of *OR22a::GFP* fluorescence intensity was performed by generating a sum intensity projection of the *OR22a*-labeled neurons and quantifying the sum fluorescence GFP intensity using the Nikon AR Image Analysis software (Nikon, Melville, NY, USA), as previously described [9]. *Repo-Gal4 > UAS-mcD8GFP*, *mz0709-Gal4* > *UAS-mCD8GFP* and anti-Draper were quantified using a standardized ROI of the outer, dorsal-medial antennal lobe membrane. The mean of the intensity averaged from each slice was used. Images are presented as the Z-stack projection through the entire brain and processed using Fiji [82].

### Single nuclei sequencing

#### Tissue collection, nuclei isolation, library preparation, and sequencing.

Central brains from either axotomized or intact flies were dissected at the age of 5 and 40 days and then placed into 1.5 mL RNase-free Eppendorf tubes, flash-frozen in liquid nitrogen, and then stored at -80°C. All dissections were performed between ZT0 and ZT4. Approximately 120–140 central brains were collected for each treatment and stored on dry ice. Nuclei isolation and snRNA-seq were performed by SingulOmics (SingulOmics Corporation, New York, USA). Briefly, tissue was homogenized and lysed with Triton X-100 in RNase-free water to isolate nuclei. The nuclei were then purified, centrifuged, resuspended in PBS with RNase Inhibitor, and then diluted to ~700 nuclei/µL. Next, standardized 10x capture and library preparation were performed using the 10x Genomics Chromium Next GEM 3’ Single Cell Reagent kit v3.1 (10x Genomics, Pleasanton, CA, USA). The prepared libraries were then sequenced using Illumina NovaSeq 6000 (Illumina, San Diego, CA, USA). The snRNA-seq raw sequencing files were processed using CellRanger 6.0 (10x Genomics, Pleasanton, CA, USA) and then mapped to the *Drosophila* reference genome (Flybase r6.31).

#### Data normalization and integration.

All analysis of snRNA-seq data was performed in R (v4.3) using the package Seurat (v5.0.2; [83]). Unless otherwise noted, default parameters were used for all operations. Each sample was independently normalized using the SCTransform function (v2;) [84,85]. After normalization, integration features were selected using the SelectIntegrationFeatures function, with nFeatures = 3000. Normalized data was prepared for integration with the PrepSCTIntegration function. Integration anchors were identified using the FindIntegrationAnchors function, with normalization.method = “SCT”. Lastly, using these anchors, the data was integrated with the IntegrateData command.

Following integration, Principal Component Analysis was performed using the RunPCA function, with npcs = 30. The resulting PCs were used to identify clusters, with a resolution of 0.2, resulting in 27 total clusters. The integrated data was prepared for marker identification by running the PrepSCTFindMarkers function, and then markers were identified with the FindAllMarkers command, using the MAST statistical test [86], which has been found to perform well on single-cell data sets [87].

The list of marker genes was used to annotate cluster identity. First, we compared the marker genes for each cluster to previously generated datasets in *Drosophila*, using the Cell Marker Enrichment tool (DRscDB; https://www.flyrnai.org/tools/single_cell/web/enrichment [88]). Wherever possible, we refined our labels by comparing marker genes with published literature marking known cell types [89].

#### Differential gene expression analysis.

To identify genes within nuclei that are differentially expressed between each of the different treatments, we applied the FindMarkers function on these cells, again using the MAST statistical test, and set group.by= ‘treatment’, separately in the you or old cohort. Genes with an adjusted *P* value less than 0.05 and an average Log_2_ Fold Change greater than 0.25 or less than -0.25 were considered differentially expressed and used for downstream analysis. To comprehensively compare gene expression within the same cell type across young and old samples under both intact and ablation conditions, as well as to validate the differential expression genes (DEGs) identified by the FindMarkers function, pseudobulk differential expression analysis was performed as needed. First, we use AggregateExpression() function to sum together gene counts of all the cells from the same sample for each cell type. This involves generating matching metadata at the sample level for each cell type. Then we normalized the pseudobulk count matrix and performed DE analysis using DESeq2 package (v1.46.0) to identify genes that are differentially expressed between experimental conditions. Variance-stabilizing transformation (VST) was applied to the count data to stabilize the variance across different expression levels and makes the data more suitable for downstream analysis. To visualize gene expressions in different patterns, we normalized the expression data by calculating z-scores for each gene across samples and then used ClusterGVis package (v0.0.2) for clustering and visualizing gene expression trends.

#### Subclustering glia clusters.

We first identified all clusters labeled as ‘Glia’ through UMAP analysis of our entire data set and extracted these cells for further analysis. Subsequently, PCA was conducted exclusively on these glial cells. The two principal components (PCs) that were found to be significant were then utilized to carry out a new round of dimensionality reduction, which was guided by Jackstraw analysis. The resolution for clustering was set at 2.5, which was determined by visually inspecting the distinct UMAP populations and ensuring that they could be clearly identified as separate clusters. Finally, differential expression analysis was performed between each of the different treatments in each glial subtype clusters, employing the same FindMarkers function as previously described.

#### Gene ontology analysis.

To identify the overarching GO terms of the resulting lists of significant ablation markers, we used clusterProfiler package (v4.8.3), as custom background, to conduct gene set enrichment analyses. The log2 fold change (log2FC) values were calculated for each gene expressed in the cell population of interest, comparing the young ablated and intact groups. The sorted log2FC list was then input into the gseGO function with the following parameters: ontology set to ‘Biological Process’ (ont = ‘BP’), gene identifier type set to ‘SYMBOL’ (keyType = ‘SYMBOL’), minimum gene set size set to 3 (minGSSize = 3), maximum gene set size set to 800 (maxGSSize = 800), organism database set to org.Dm.e.g.,db (OrgDb = org.Dm.e.g.,db), and p-value cutoff set to 0.01 (pvalueCutoff = 0.01). GO terms were subsequently grouped according to parent terms based on semantic similarity as calculated using the mgoSim() function of the GOSemSim R package (v.2.20.0) and the reduceSimMatrix() function in the rrvgo package (v.1.6.0).

### Statistical analysis

All data of sleep duration and florescence intensity are presented as box plots with ± SEM error bar. Unless stated otherwise, a one-way analysis of variance (ANOVA) was used for comparisons involving two or more genotypes under a single treatment, while a two-way ANOVA was employed for comparisons involving multiple genotypes subjected to two treatments. Post hoc analyses were conducted using Sidak’s multiple comparisons test. Each experiment was performed with a minimum of two independent runs, and the sample size for each genotype and treatment combination is represented either as individual points or specified in the corresponding figure legends. All statistical analyses were performed using InStat software GraphPad Prism (version 10.3.0 for Windows, Boston, Massachusetts, USA), where N denotes the number of biological samples tested.

## Supporting information

S1 Data (XLSX)

S1 Fig Reduced pan-glial membrane infiltration after neuronal injury in aged flies. S1G Fig created in BioRender. Keene, A. (2025) https://BioRender.com/ejp67b0.
(PDF)

S2 Fig Age-dependent changes in sleep architecture following neuronal injury. (PDF)

S3 Fig Sleep is dysregulated in Draper-deficient flies. (PDF)

S4 Fig Gaboxadol-induced sleep enhances glial-mediated clearance in 5-day-old flies. S4A Fig created in BioRender. Keene, A. (2025) https://BioRender.com/ejp67b0.
(PDF)

S5 Fig Genetically induced sleep alters sleep architectures in 5-day- and 40-day-old flies upon 23E10 neuron activation. S5A Fig created in BioRender. Keene, A. (2025) https://BioRender.com/ejp67b0.
(PDF)

S6 Fig snRNA-seq of central brains in young and aged intact or ablated flies. (PDF)

S7 Fig DEG clustering via pseudobulk analysis for olfactory ensheathing glia cells. (PDF)
