# Supplementary figures and images for: Sleep rescues age-associated loss of glial engulfment
Source: PLoS Genet. 2026 Jan 13;22(1):e1011999. doi: 10.1371/journal.pgen.1011999 (PMC12858066; doi:10.1371/journal.pgen.1011999)

Supplementary Figure 1

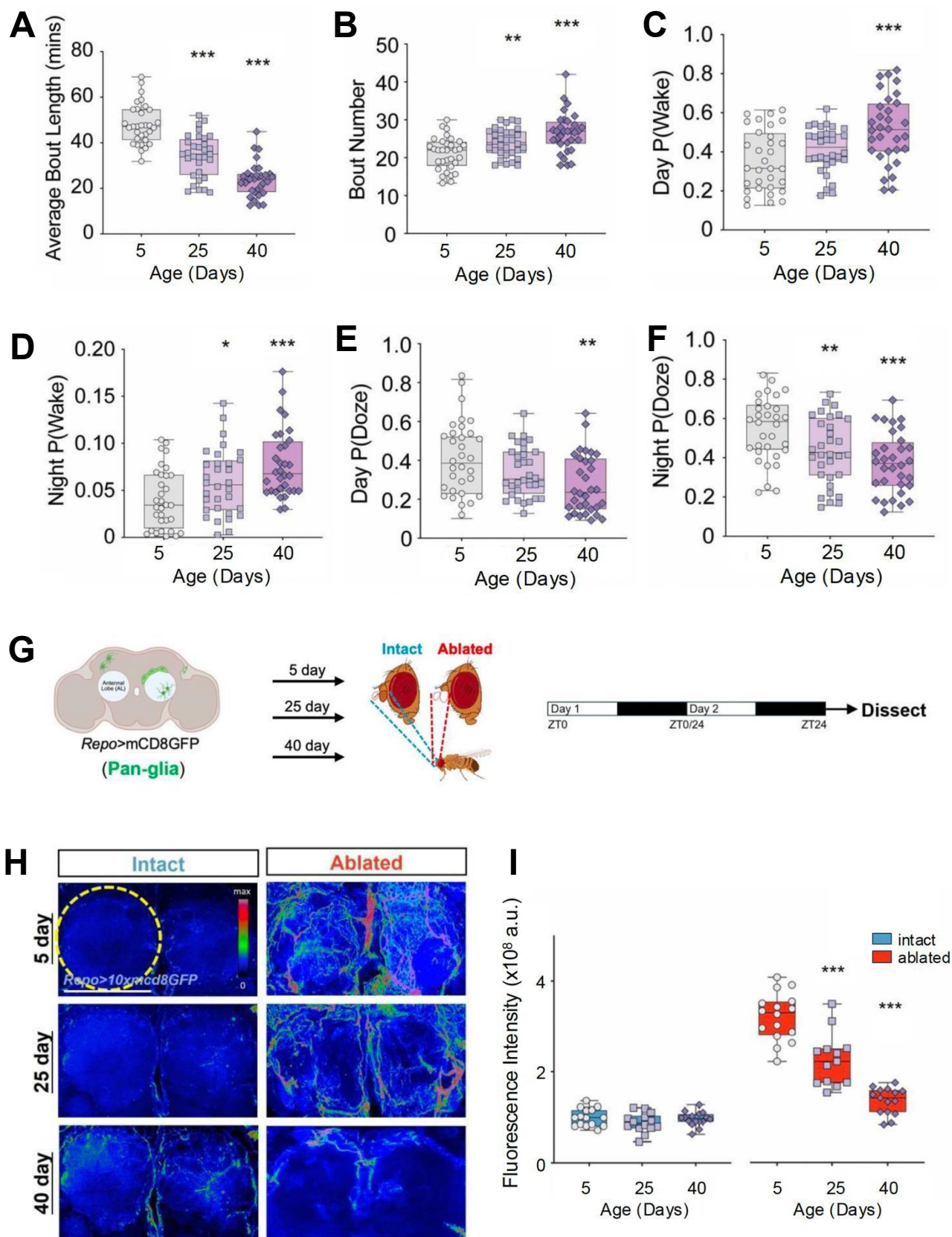

Supplement: S1 Fig — S1G Fig created in BioRender. Keene, A. (2025) https://BioRender.com/ejp67b0. (PDF) [file pgen.1011999.s002.pdf]

Supplementary Figure 2

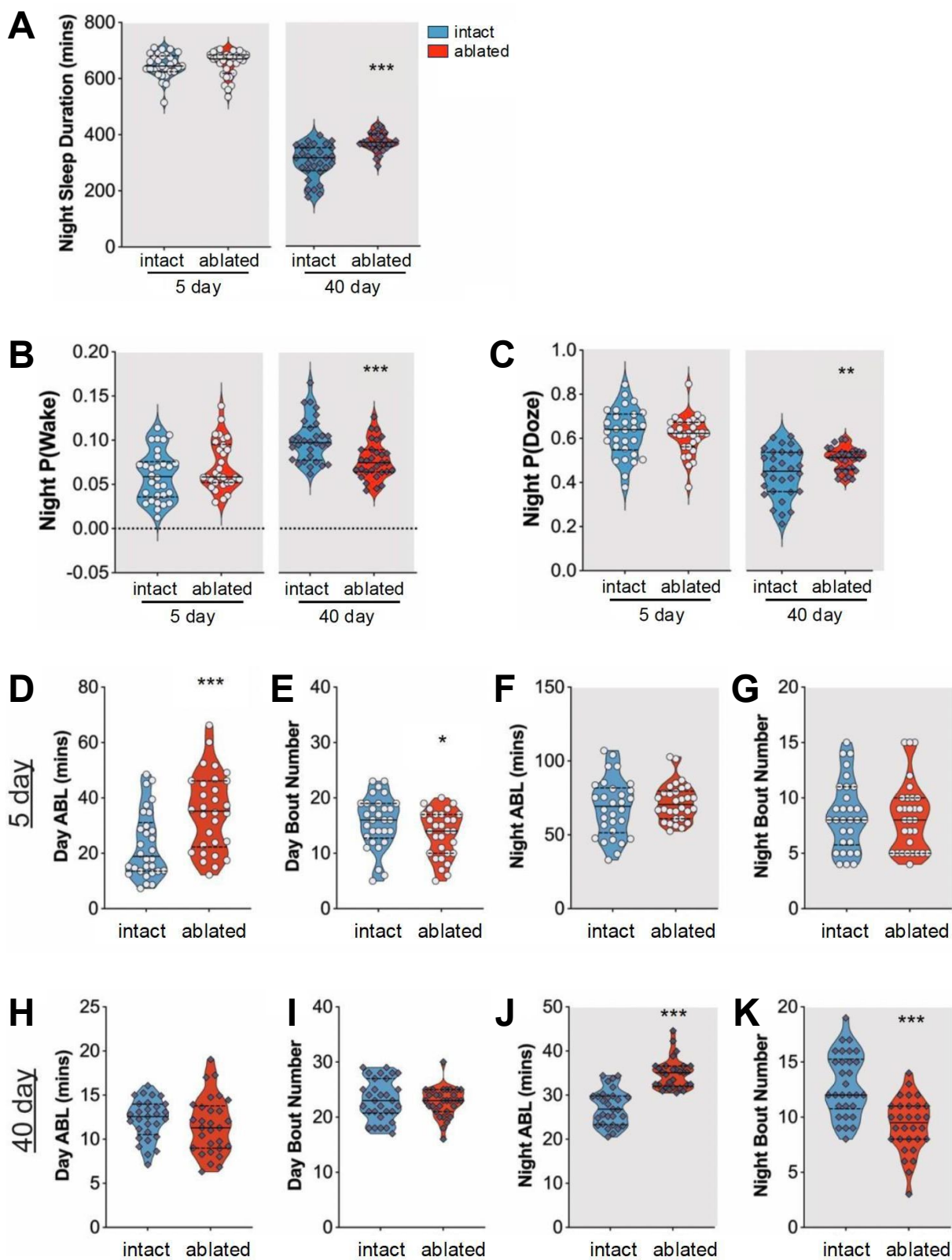

Supplement: S2 Fig — (PDF) [file pgen.1011999.s003.pdf]

**A**

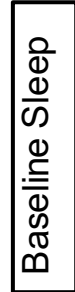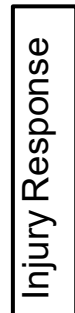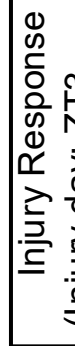

Supplement: S3 Fig — (PDF) [file pgen.1011999.s004.pdf]

Supplementary Figure 4

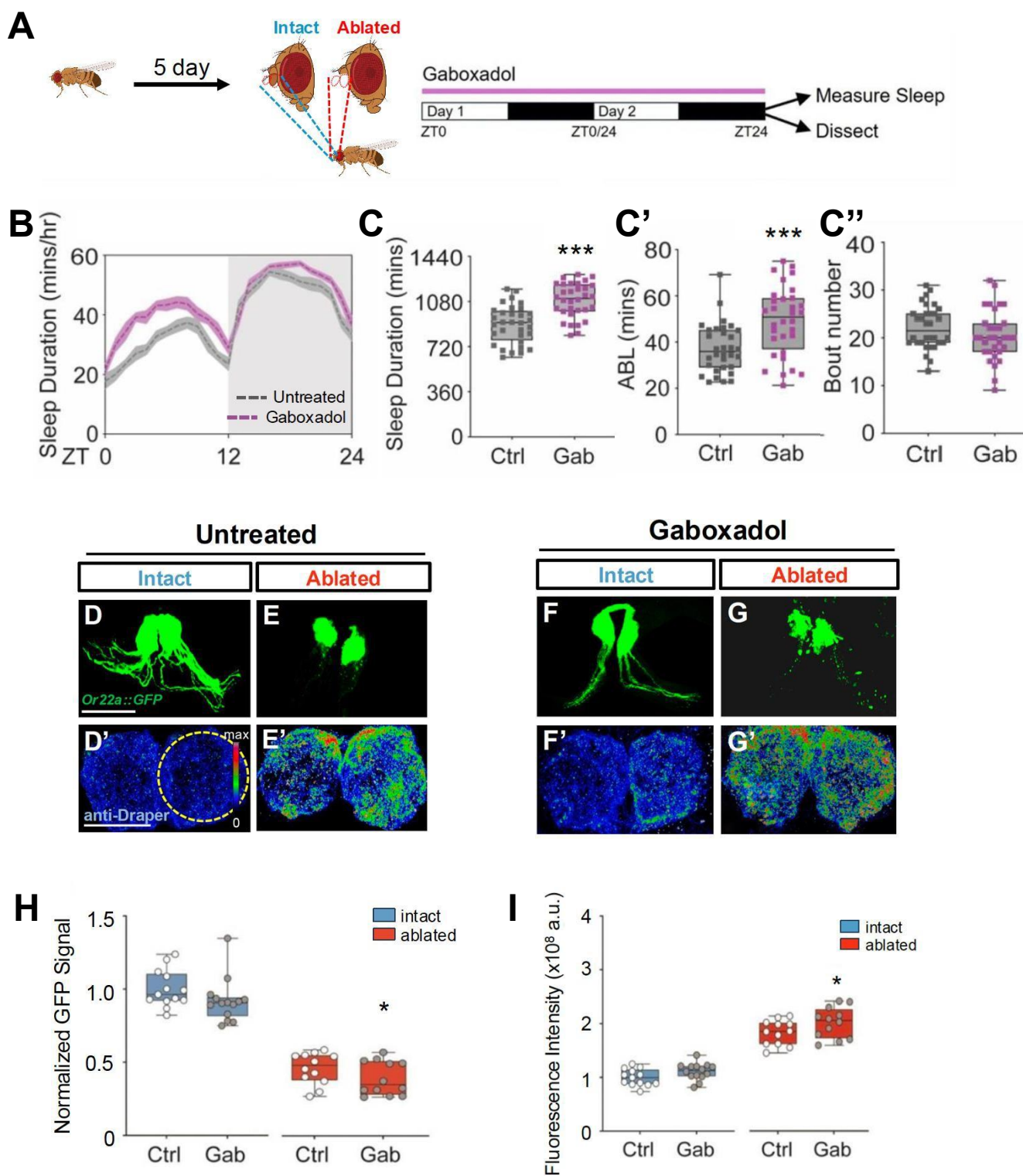

Supplement: S4 Fig — S4A Fig created in BioRender. Keene, A. (2025) https://BioRender.com/ejp67b0. (PDF) [file pgen.1011999.s005.pdf]

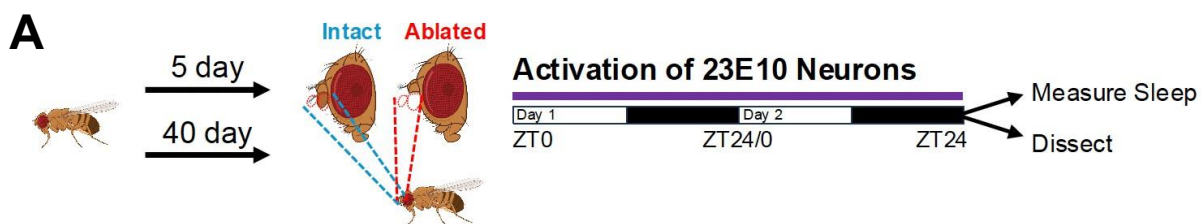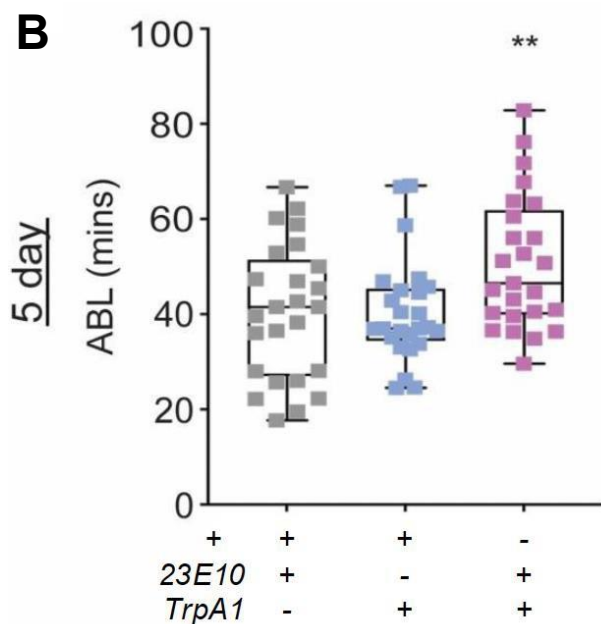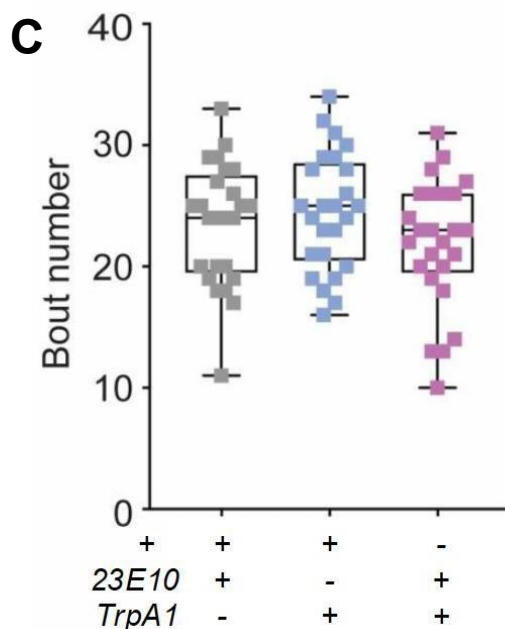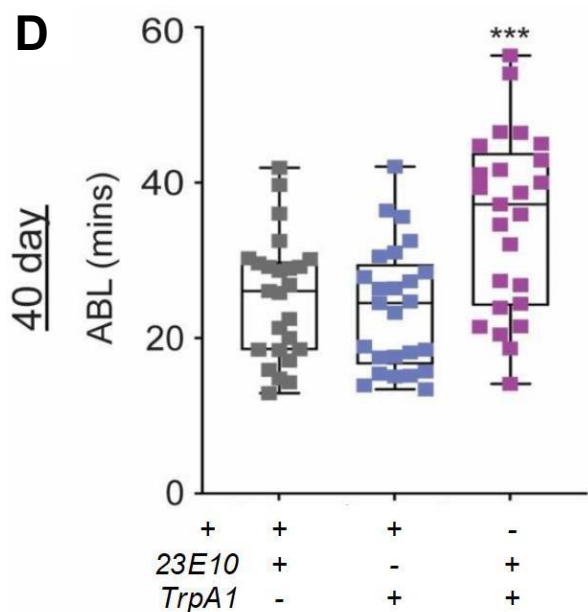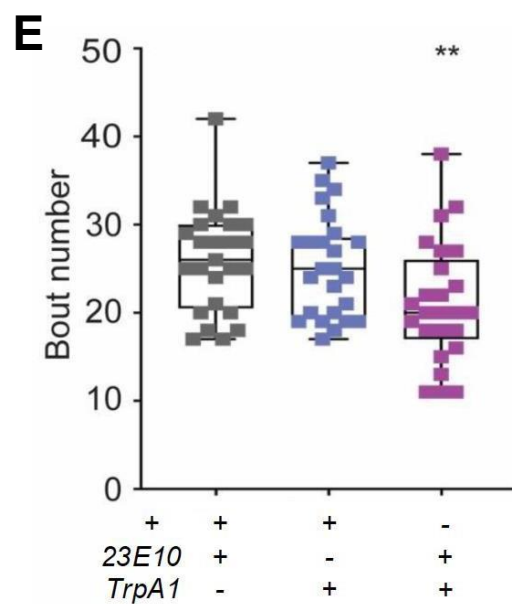

Supplement: S5 Fig — S5A Fig created in BioRender. Keene, A. (2025) https://BioRender.com/ejp67b0. (PDF) [file pgen.1011999.s006.pdf]

Supplementary Figure 6

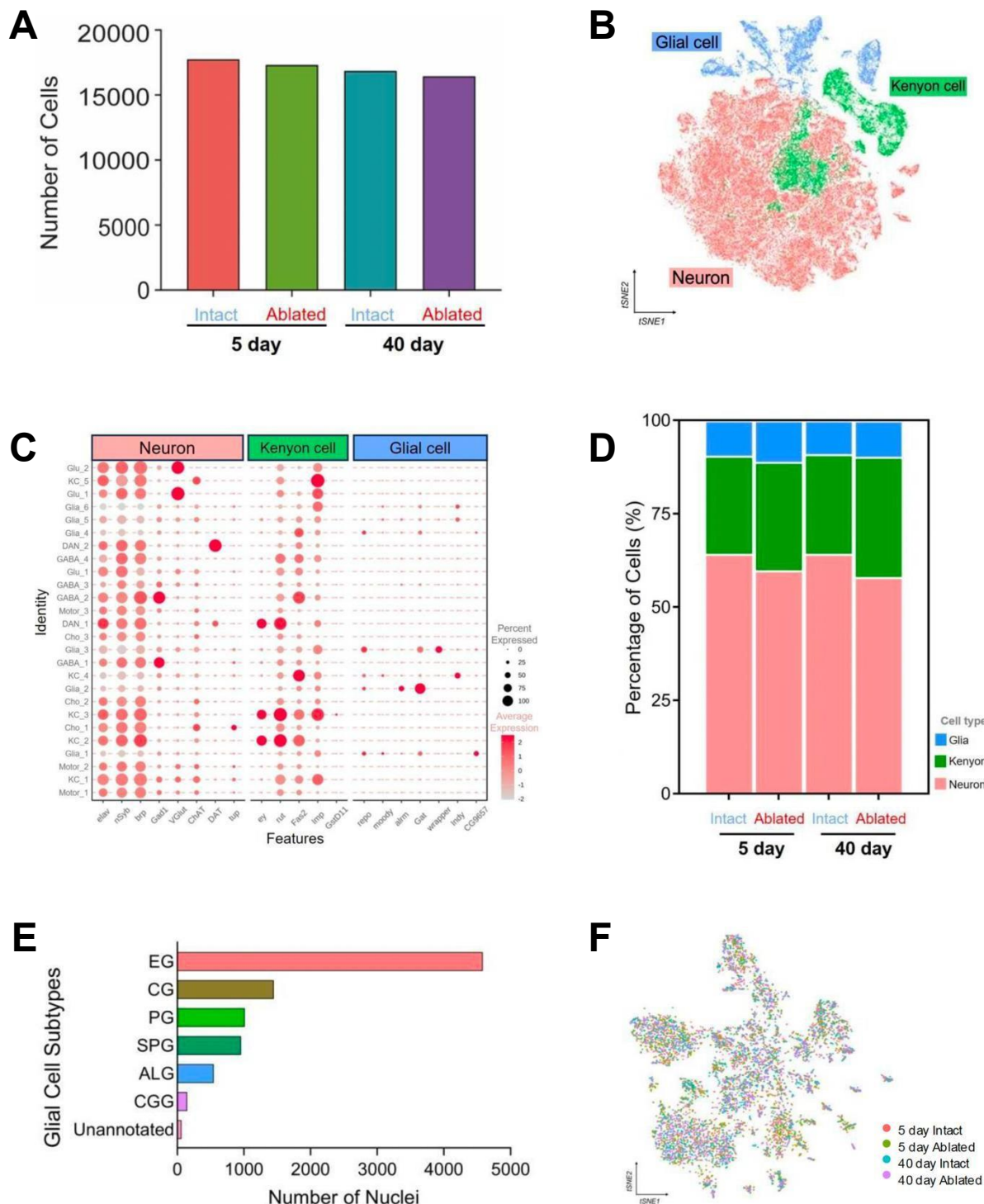

Supplement: S6 Fig — (PDF) [file pgen.1011999.s007.pdf]

Supplementary Figure 7

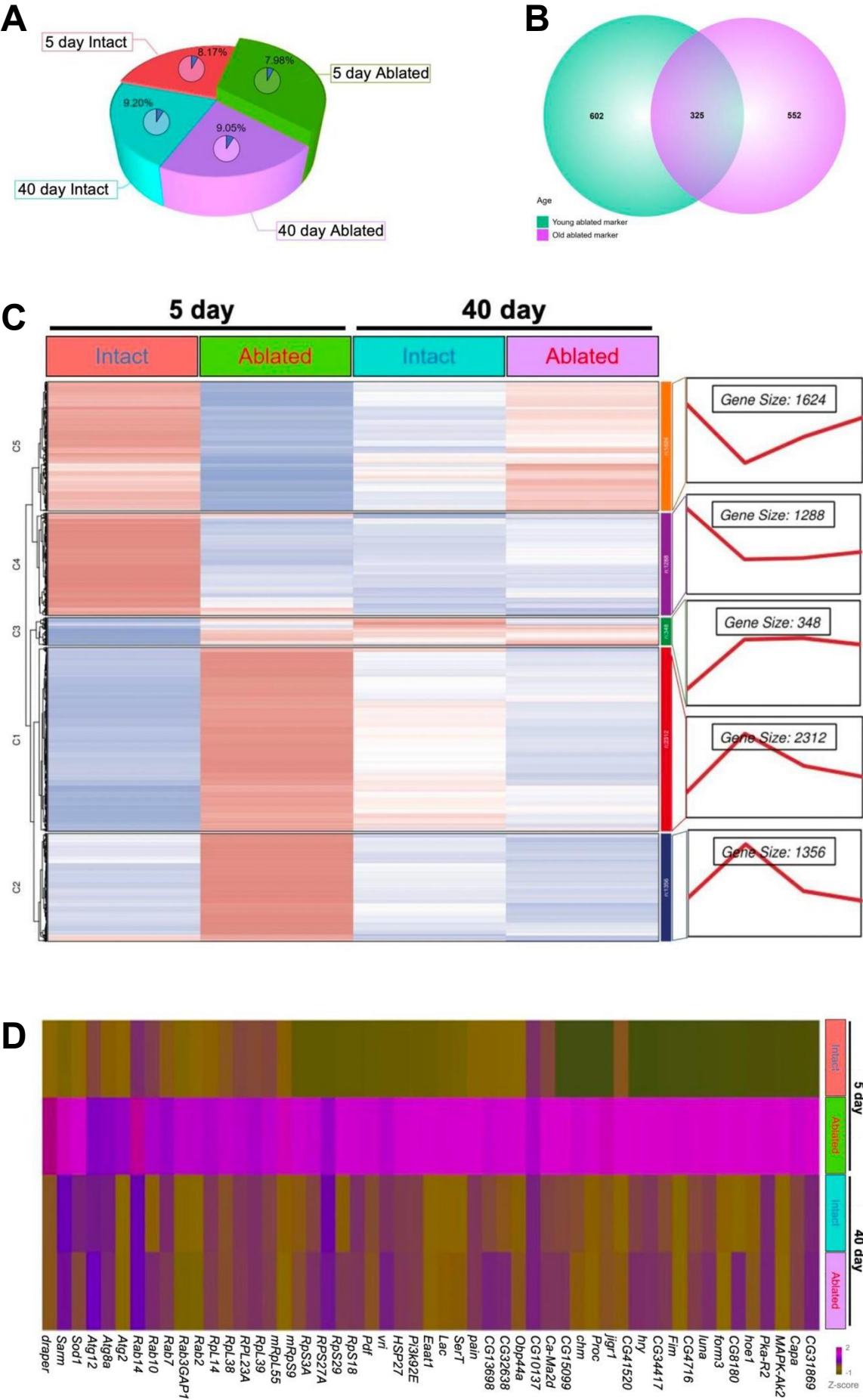

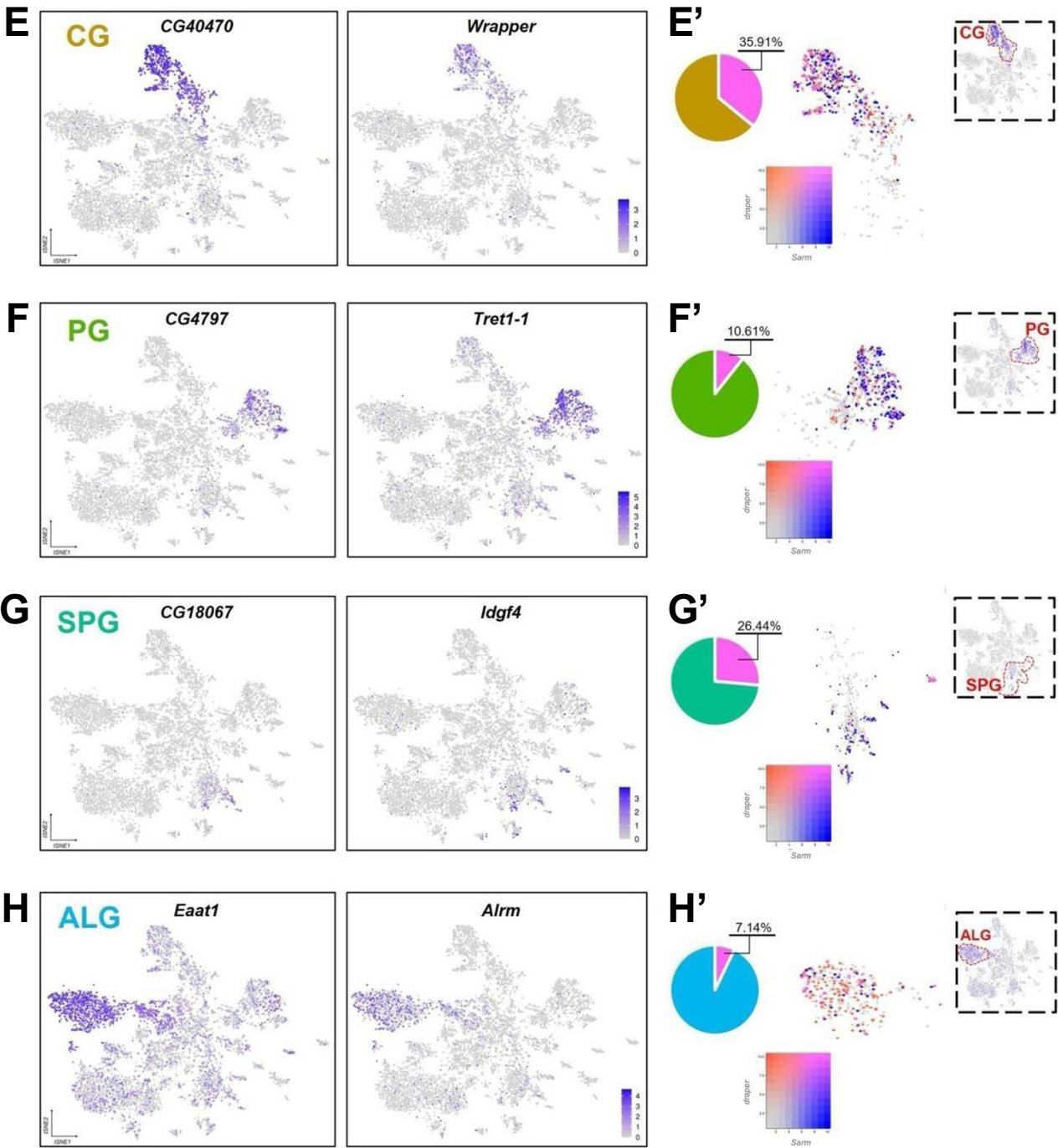

Supplement: S7 Fig — (PDF) [file pgen.1011999.s008.pdf]
